# Synthesis and Physicochemical Evaluation of Bees’ Chitosan-Based Hydrogels Modified with Yellow Tea Extract

**DOI:** 10.3390/ma14123379

**Published:** 2021-06-18

**Authors:** Sonia Kudłacik-Kramarczyk, Anna Drabczyk, Magdalena Głąb, Paweł Gajda, Anna Jaromin, Anna Czopek, Agnieszka Zagórska, Bożena Tyliszczak

**Affiliations:** 1 Department of Materials Science, Faculty of Materials Engineering and Physics, Cracow University of Technology, 37 Jana Pawła II Av., 31-864 Krakow, Poland; 2 Department of Nuclear Energy, Faculty of Energy end Fuels, AGH University of Science and Technology, 30 Mickiewicza Av., 30-059 Krakow, Poland; pgajda@agh.edu.pl; 3 Department of Lipids and Liposomes, Faculty of Biotechnology, University of Wrocław, 14a Joliot-Curie St., 50-383 Wrocław, Poland; anna.jaromin@uwr.edu.pl; 4 Department of Medicinal Chemistry, Faculty of Pharmacy, Jagiellonian University Medical College, 9 Medyczna St., 30-688 Krakow, Poland; anna.czopek@uj.edu.pl (A.C.); agnieszka.zagorska@uj.edu.pl (A.Z.)

**Keywords:** hydrogel polymers, yellow tea extract, wettability measurements, strength properties, swelling ability, degradation in physiological liquids

## Abstract

The novelty of the research involves designing the measurement methodology aimed at determining the structure–property relationships in the chitosan-based hydrogels containing yellow tea extract. Performed investigations allowed us to determine the swelling properties of hydrogels in selected time intervals, evaluate the mutual interactions between the hydrogels and simulated physiological liquids via pH measurements and directly assess the impact of such interactions on the chemical structure of hydrogels using Fourier transform infrared (FT-IR) spectroscopy and their wettability by the measurements of the flatness of the drop on the surface of the tested samples via the static drop method. Next, the surface morphology of hydrogels was characterized by the Scanning Electron Miscorcopy (SEM) and their elasticity under the tension applied was also verified. It was proved that incubation in simulated physiological liquids resulted in a decrease in contact angles of hydrogels, even by 60%. This also caused their certain degradation which was reflected in lower intensities of bands on FT-IR spectra. Further, 23% *v/v* yellow tea extract in hydrogel matrices caused the decrease of their tensile strength. An increase in the amount of the crosslinker resulted in a decrease in the sorption capacity of hydrogels wherein their modification caused greater swelling ability. In general, the investigations performed provided much information on the tested materials which may be meaningful considering their application, e.g., as dressing materials.

## 1. Introduction

Hydrogels belong to the group of polymers with growing interest in many fields including medicine and the relative areas [1,2,3]. Their popularity results from many unique properties including, e.g., the ease of modification with compounds of various origin and properties [4,5,6]. Considering the application possibilities, determining the structure–property relationships in hydrogels allows for modification that leads to the preparation of materials with desirable features [7,8]. The measurement methodology applied depends on many factors including the potential use of such materials, the form of the material tested or its chemical composition (i.e., the type of hydrogel matrix or the modifying agent). For example, there are many methods that may be applied to characterize the mechanical properties of hydrogels, e.g., ring extensiometry, strip extensiometry, compression test, indentation test or bulge test. Nonetheless, these analyses are destructive and in the case of the need to use the non-destructive method (i.e., in tissue engineering applications) the approaches including the online monitoring are performed [9]. Other important properties determining the use of hydrogels are their swelling abilities. For example, Syed et al. distinguished swelling rate measuring methods based on determining the free-absorbency capacity including the tea-bag method, the filtration method or the sieve method depending on the desired precision and the amount of the hydrogel sample available for the analysis. Moreover, in the paper, another method is developed, i.e., the sieve filtration method, with higher efficiency, repeatability and reproducibility [10]. On the other hand, Sievers et al. reported two main approaches allowing to determine the volume expansion of hydrogels resulting from the swelling. The first approach concerns the multiple scaling of the hydrogel before and after sorption followed by the calculation of the swelling ratio using the solvent and the polymer density. The second method is based on the direct measurements of hydrogel dimensions in the initial and swollen state followed by the calculation of the swelling ratio using the data obtained [11].

Considering the modification of hydrogels, growing attention is directed toward additives of natural origin such as plant extracts. They are particularly promising for application for biomedical purposes due to their rich chemical composition [12]. For example, one of the most interesting components of plant extracts is rosmarinic acid characterized by antioxidant activity [13]. Furthermore, plant extracts exhibit antimicrobial activity [14,15,16]. Manilal et al. described in vitro antibacterial activity of *Rosmarinus officinalis* and *Moringa stenopetala* extracts against *Staphylococcus aureus*. This bacterial strain is methicillin-resistant and new approaches to dealing with such bacteria are highly desired [17]. Next, Sabo et al. reported that *Eucalyptus camaldulensis* extract exhibited antibacterial activity both against Gram-negative and Gram-positive bacteria [18]. Additionally, there are many reports proving that plant extract may reduce the blood glucose level [19]. Abdel-Lateff et al. proved that extract from *Euryops arabicus* (*Asteraceae*) exhibited antioxidant activity and, importantly, anti-inflammatory properties [20].

Yellow tea extract is one of the extracts with a rich chemical composition [21]. Many performed investigations proved that yellow tea consists of such compounds as polyphenols (e.g., catechins or flavone glycosides), amino acids or methylxanthines [22]. All these substances contribute to the properties such as antioxidative [23,24], anticancer [25,26] or anti-inflammatory ones [27] showed by yellow tea. These features make this extract useful as a modifying agent of various materials considered for biomedical use, e.g., hydrogels. These polymers show biocompatibility, non-toxicity and great swelling capability [28,29,30]. These features in combination with such modifiers as plant extracts contribute to the preparation of materials with great application potential in medicine. For example, Elegbede et al. carried out studies on the hydrogels modified with extract from *Aspalathus linearis* (rooibos) considering their use for surgical wound healing [31]. Among the groups of biopolymers widely used for the synthesis of hydrogels is chitosan, which shows antimicrobial activity toward fungi and both Gram-positive and Gram-negative bacteria [32,33].

Antimicrobial activity of chitosan in combination with such features as, e.g., biocompatibility, antitumor and immunomodulatory activity or biodegradability, make this biopolymer widely applied for biomedical purposes [34,35] including drug or gene delivery [36,37], tissue engineering [38] or wound healing [39,40]. In turn, the combination of chitosan and natural plant extracts due to the beneficial pharmacological properties of these two components leads to the synthesis of hydrogels promising for biomedical use. For example, Ragab et al. proposed chitosan-based hydrogels with extract from *P. granatum* peel as materials for application in chronic wound treatment [41]. Pankongadisak et al. used longan seed extract for modification of hydrogels based on chitosan, also containing silk fibroin which was subsequently characterized as a material with application potential in bone tissue engineering [42].

Here, synthesis and investigations on hydrogels based on bees’ chitosan modified with yellow tea extract are presented. The materials obtained were analyzed from the viewpoint of their potential application as innovative dressing materials. Such hydrogel dressings, due to their ability to absorb various liquids, may absorb the wound exudate and—importantly—may also show therapeutic properties due to the possibility of introduction into their matrix various bioactive substances. Studies on hydrogel materials as potential dressings were described also by Nurzynska et al. [43] and Jin et al. [44]. The presence of tea extract may enhance the polymers with additional therapeutic properties beneficial in the viewpoint of the wound healing process which was proved in the research of Wang et al. [45].

According to our knowledge, any measurement methodology aimed at determining the structure–property relationships in the chitosan-based hydrogels modified with yellow tea extract has not been presented. Furthermore, the synthesis methodology proposed is based on the crosslinking via UV radiation the reaction mixture containing Beetosan^®^, i.e., chitosan obtained from naturally deceased bees. In general, the preparation of Beetosan^®^ involved a multistep treatment of honeybees including removal of waxes, mineral salt, proteins and, finally, pigments. The methodology of its preparation has been described in more detail in [46]. Apart from Beetosan^®^, gelatin was also used for the synthesis of hydrogel matrices due to the fact that according to some literature reports it was proved that this biopolymer provides the material with elasticity [47]. The methodology applied allows determining the swelling properties of hydrogels but also discussion of the behavior of drop of water during the first contact with hydrogel surfaces, i.e., determining the wettability of obtained materials. Performed studies also included the analyses of the mutual interactions between the hydrogels and simulated physiological liquids and the impact of such environments on polymers’ wettability. Next, conducted analyses also allowed a direct evaluation of the impact of incubation in simulated physiological liquids on the chemical structure of hydrogels. Finally, attention was also paid to determining the elasticity of hydrogels under the tension applied.

## 2. Materials and Methods

### 2.1. Materials

All chemicals used in the research were at least of analytical reagent grade. The gelatin was received from Avantor Performance Materials, Gliwice, Poland. Chitosan (deacetylation degree 75–85%, low molecular weight 50,000–190,000 Da), 2-hydroxy-2-methylpropiophenone (photoinitiator; 97%, d = 1.077 g/mL; Mw = 164.20 g/mol) as well as poly(ethylene glycol) diacrylate (crosslinking agent, Mn = 700 g/mol) was bought in Merck (Darmstadt, Germany). The Kekecha yellow tea was purchased in Camellia Sinensis store. Beetosan^®^ was received as a result of multistep chemical treatment of naturally deceased honeybees including removal of waxes, mineral salts, proteins and pigments. The process of the preparation of Beetosan^®^ from honeybees has been previously described in Ref. [46].

### 2.2. Preparation of Kekecha Yellow Tea Extract

**Figure 1:**
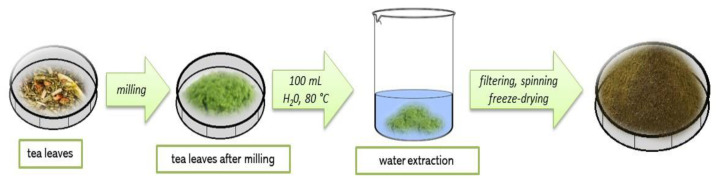
The scheme of the preparation of tea lyophilizate.

Firstly, 10 g of tea leaves were ground in a mill to a fine powder. Next, water extraction was performed by pouring the powder obtained with 100 mL of water at 80 °C and then the mixture was left for 15 min. Extraction was conducted twice, the amount of water used was reduced by half in the second case and the infusion was filtered on a corrugated filter. The filtrate was centrifuged for 15 min (2700 rpm), the supernatant liquid was decanted and obtained precipitate was lyophilized. The powder after lyophilization was stored in a cool, dry and shady place. The scheme of the described procedure is shown in Figure 1.

Yellow tea extract was prepared as follows: 2 g of lyophilized powder was treated with 50 mL of distilled water and the suspension was boiled for 15 min. After this period, yellow tea extract was applied for modification of hydrogel materials.

### 2.3. Synthesis of Hydrogels Modified with Yellow Tea

Firstly, Beetosan^®^ and gelatin were dissolved in 0.05% acetic acid solution (such obtained mixture was defined as a base solution). Concentration of Beetosan^®^ was 3% and gelatin 2%, respectively. Next, yellow tea extract, crosslinking agent and photoinitiator were added to the previously prepared base solution, the whole mixture was poured down on a Petri dish and treated with UV radiation for 2 min. As a source of radiation, EMITA VP-60 lamp (Famed, Lodz, Poland) (λ = 320 nm, power: 180 W) was used. In Table 1, the compositions of hydrogel materials obtained are presented.

**Figure 2:**
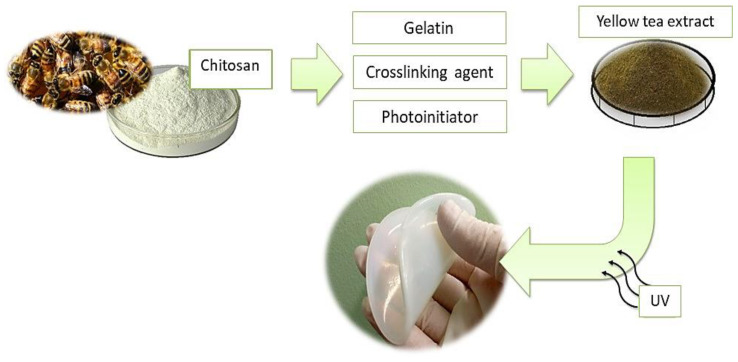
The scheme of the hydrogels’ preparation.

In Figure 2. scheme of the synthesis of hydrogel materials is presented.

Prepared samples were immersed in phosphate-buffered saline (PBS) for 15 min to remove possible unreacted reagents and then dried at 40 °C for 24 h. Next, they were cut into small samples (approx. 1.0 g) and subjected to further research.

### 2.4. Methodology of Measurements

#### 2.4.1. Characterization of the Sorption Properties of Hydrogels

The methodology of the swelling sorption measurements was as follows: hydrogel samples (mass: approx. 1.0 g, diameter: 20 mm, thickness: 0.1 mm) were dried for 24 h at room temperature. Then, they were weighed and introduced into the sterile vessels containing 50 mL of the adequate liquid, i.e., distilled water, simulated body fluid (SBF, solution isotonic to the human blood plasma [48], Ringer liquid (infusion liquid consisting of potassium chloride, calcium chloride and sodium chloride use, e.g., to restore the chemical balance during the isotonic dehydration treatment [49]) or artificial saliva solution. The study was performed at room temperature to check the sorption ability of obtained materials without affecting this process via the temperature. After 1 h of an immersion analyzed samples were separated from the mentioned liquids, the excess surface water was removed via paper towels (to determine only the amount of liquid bound with the tested hydrogel) and the swollen samples were weighed again. Subsequently, they were introduced into the liquids again and the study was performed analogously. Mass of samples was verified also after 24, 48 and 120 h. The sorption capability of the hydrogels was measured using the following equation:

(1) $$Q=\frac{m-m_{0}}{m_{0}},$$

where:

Q—swelling ratio, g/g; 

m—weight of swollen sample, g; 

$m_{0}$—weight of dry sample, g.

Many investigations were performed to evaluate the swelling capacity of hydrogels which determine their wide area of potential applications. Notably, it is important to select an adequate methodology to evaluate these properties, e.g., the temperature conditions. It was proved by Shah et al. or Kipcak et al. that the swelling capability of hydrogels increases with the temperature increase which is probably caused by the increase in the mobility of polymer chains. This, in turn, facilitates the expansion of the polymer network and makes more space for water [50,51]. It is also important to determine the swelling properties of hydrogel polymers in specific environments, i.e., the ones in which the application of such materials is considered. Compositions of absorbed solutions, i.e., various ions or any other atoms, may also affect the hydrogels’ absorbency via interactions with functional groups or ions formed as a result of dissociation. Other factors affecting the hydrogels’ swelling are, e.g., electric field, ionic strength or even light [52].

#### 2.4.2. Evaluation of the Mutual Interactions Between Hydrogels and Simulated PhysioLogical Liquids

The next investigations involved the evaluation of mutual interactions between the hydrogels and the environments simulating human physiological liquids. For this purpose, hydrogel samples (mass: approx. 1.0 g, diameter: 20 mm, thickness: 0.1 mm) were placed in sterile vessels containing 50 mL of previously mentioned liquids. The vessels were tightly closed and placed in a laboratory incubator at a temperature of 37 °C. Such selected methodology allowed us to simulate conditions similar to these one occurring in the human body and to limit the impact of the external environment (due to the tight closure of the vessels). Performed studies based on the pH measurement of the liquids every two days via the multifunction pH-meter Elmetron CX-701 (Zabrze, Poland). The study was performed in distilled water, SBF solution, Ringer liquid and artificial saliva solution. Analyzing swelling properties of hydrogels, the attention was paid only to determining the capability of samples to absorb selected liquids in specific time intervals—the other interactions between the polymer and the tested medium were not considered. Here, attention is paid to the mutual impact of long-term immersion (i.e., 21 days) on the liquids and on tested hydrogels. The incubation of hydrogel samples in tested liquids may contribute, e.g., to the elution of all non-crosslinked reagents, i.e., both reagents forming a hydrogel matrix, crosslinker, photoinitiator or additive. Next, specific conditions of the immersion, i.e., the chemical compositions and various pH of selected fluids, may result in the degradation of immersed samples. On the other hand, various ions or other molecules included in the compositions of the incubation media also may interact with analyzed polymers. Due to the fact that some of these phenomena may result, e.g., in the change of pH of the incubation liquid, a methodology based on the regular pH measurements was designed.

#### 2.4.3. Analysis of the Chemical Structure of Hydrogels via FT-IR Spectroscopy

Fourier transform infrared (FT-IR) spectroscopy was performed to discuss the direct impact of the incubation of hydrogels in SBF on their chemical structure, i.e., the chemical bonds or the functional groups present in the crosslinked hydrogel structure. This method allows identifying the specific chemical structures due to their ability to vibrate under the influence of infrared light absorption wherein various structures vibrate under the light of a different wavelength from this range. The study was performed for samples before and after incubation in SBF solution. Samples (mass: approx. 1.0 g, diameter: 20 mm, thickness: 0.1 mm) before incubation were dried at room temperature for 24 h and then subjected to the analysis. Samples after 21-days of incubation in SBF were separated from the solution, the excess surface water was removed by the paper towel and they were dried at room temperature for 24 h. The analysis was performed at room temperature using the Thermo Scientific Nicolet iS5 equipped with ATR diamond accessory (Loughborough, UK). The spectra were recorded in the range of 4000–500 cm^−1^ (32 scans at 4.0 cm^−1^ resolution).

#### 2.4.4. Measurements of the Surface Contact Angles of Hydrogels

Apart from the interactions which take place between hydrogel samples and absorbed liquids causing the hydrogel swelling it is also important to evaluate the behavior of the drop of the tested liquid during the first contact with hydrogel surface. Therefore the next investigations included the analysis of hydrogels’ wettability, i.e., the evaluation of how much the drop of liquid spreads over the hydrogels’ surface. Such behavior is determined via the measurements of the flatness of the drop on the surface of the tested sample. This, in turn, is determined, e.g., via the static drop method and such a methodology was applied in the described investigations.

The study was performed for samples before and after 21-days of incubation in SBF solution which was prepared for the measurements in the same way as it was described in Section 2.4.3. Analyses were carried out using Kruss DSA 100M (Hamburg, Germany) at room temperature and in the air atmosphere. Samples were placed in the apparatus chamber and the drop of water (10 μL) was dosed on the sample’s surface via a micro-pipette (micro-syringe). The investigation was accompanied by a video recording and the contact angles were determined from the video images via fitting the polynomial curves to the surface of the tested sample and the geometry of the drop of liquid.

#### 2.4.5. SEM Analysis of Hydrogel Surfaces

The wettability of samples depends, e.g., on the surface morphology therefore subsequent analyzes included characterization of the surface topography via scanning electron microscopy (SEM). The analysis was performed using Helios NanoLabHP FEI Electron Microscope (Hillsboro, OR, USA). Before the measurements, hydrogel samples (mass: approx. 1.0 g, diameter: 20 mm, thickness: 0.1 mm) were dried at room temperature for 24 h, placed in a holder and treated with gold to form a coating (thickness: approx. 10 nm). SEM analyses were performed at room temperature.

#### 2.4.6. Analysis of the Tensile Strength of Hydrogels

The investigations performed included also determining the behavior of the materials under the tension applied. The study was performed at room temperature using the Brookfield CT3 texture analyzer (Middleboro, MA, USA). The whole procedure proceeded according to the standards ISO 527-2 type 5A and ISO 37 type 2. Before experiments, hydrogel samples were dried at room temperature for 24 h. Next, the ZCP020 Manual Cutting Press was applied to form the paddle-shaped samples (with dimensions: length—30.0 mm, width—3.0 mm, depth—1.5 mm). They were next clamped between the jaws. During the analysis, the sliding apart of the jaws takes place which results in the sample stretching. The study is performed until the sample ruptures. Such a methodology provides information on the tensile strength of the hydrogels as well as on their elasticity and allows to compare the elongation of samples under the same tension.

## 3. Results and Discussion

### 3.1. Synthesis of Hydrogels

The synthesis methodology selected for the preparation of hydrogels was the photopolymerization process. This is based on the use of UV light to decompose the photoinitiator into free radicals, i.e., the active atoms or molecules, able to initiate the polymerization process. Such a methodology allows to obtain hydrogels in a short time, shows a low energy demand and there is no need to use toxic organic solvents. Moreover, there are not any side products and the hydrogel after the photopolymerization process may be subjected to further investigations.

**Figure 3:**
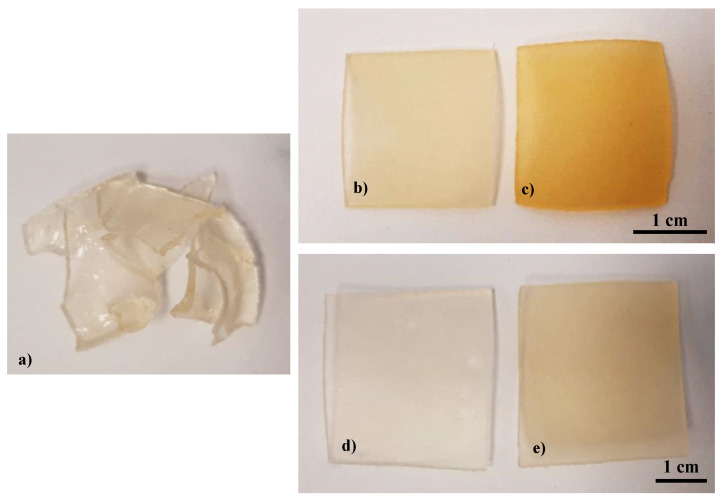
Images of obtained samples: non-crosslinked hydrogel—sample 33/3.2 (**a**); properly crosslinked unmodified sample 0/1.6 in a dry state (**b**) and in a swollen state (**d**); properly crosslinked modified sample 9/1.6 in a dry state (**c**) and after swelling (**e**).

Based on the performed syntheses, it was observed that in some cases crosslinked hydrogel sample was not obtained as a result of 2 min treatment with UV radiation. Such a conclusion was drawn in the case of the reaction mixture containing 33% *v/v* yellow tea extract and 3.2 mL of the crosslinker (sample 33/3.2) which was not crosslinked during the reaction. It resulted probably from an inadequate proportion of the reaction mixture (i.e., 10 mL of a base solution and 5 mL of the tea extract) to the amount of crosslinker applied—a larger amount of the additive was used compared to its content in the previous reaction mixtures and as a result, the amount of crosslinker applied was not sufficient to obtain crosslinked hydrogel polymer. The difference between properly crosslinked hydrogel and non-crosslinked one is presented in Figure 3. Additionally, near properly crosslinked hydrogels, their samples in a swollen state (15-min swelling) are shown.

As it may be seen, inadequate amounts of the reagents applied resulted in the preparation of non-crosslinked hydrogel samples. Next, differences between dry and swollen samples may be visible. However, more information concerning the swelling measurements is provided in the next subsection of this paper.

### 3.2. Results of Investigations on the Sorption Properties of Hydrogels

**Figure 4:**
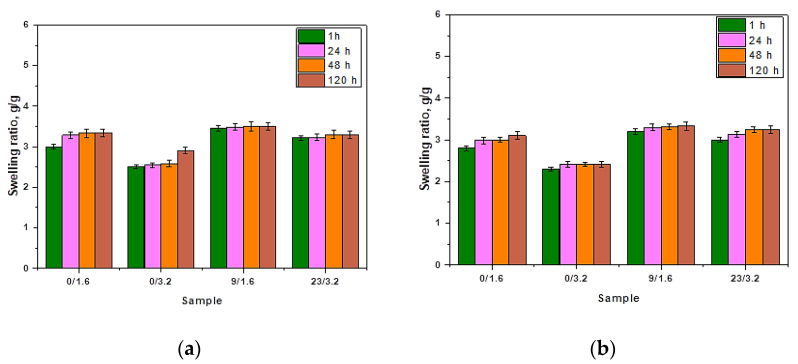

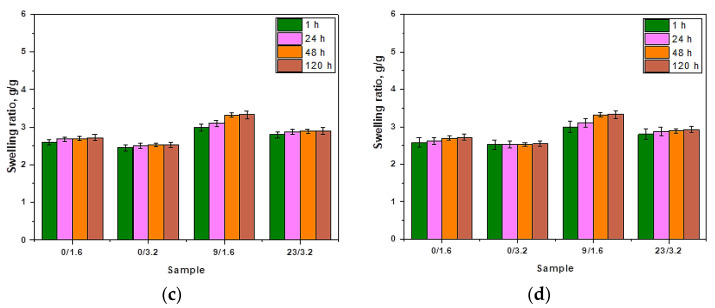
Results of swelling studies of the hydrogels in (**a**) distilled water (average value $\bar{SD}=1.20\%$, number of repetitions *n* = 3); (**b**) Ringer liquid (average value $\bar{SD}=1.40\%$, number of repetitions *n* = 3); (**c**) artificial saliva solution (average value $\bar{SD}=1.30\%$, number of repetitions *n* = 3); and (**d**) in SBF (average value $\bar{SD}=1.15\%$, number of repetitions *n* = 3).

The methodology of sorption measurements provided information on the swelling ability of hydrogels in selected liquids with various compositions and at room temperature. Tested hydrogels differed in the chemical compositions but their size, thickness and synthesis and drying conditions were the same. The results of swelling investigations are shown in Figure 4.

Firstly, it should be indicated that analyzed polymers showed sorption properties. The methodology applied enabled us to observe the differences of hydrogels’ swelling in media differing significantly in the chemical compositions. Among all calculated swelling ratios, the highest ones were calculated for samples swelling in distilled water. The difference was not significant, but noticeable. Dry hydrogels take the form of a polymer ball, in which polymer chains are packed thus showing little free space between them. As a result of the immersion of the hydrogel matrix in distilled water or any other physiological liquid, the coiled polymer structure loosens. This is due to the occurrence of the dissociation and solvation of the functional groups present in the structure of the superabsorbent. As a result of the dissociation of functional groups, ions are formed. They, in turn, repel as a result of electrostatic interactions which causes the separation of coiled polymer. In such a way, free spaces between polymer chains are formed which enables liquid penetration. Atoms of water molecules due to their polarity (i.e., asymmetrical charge distribution) have slight charges which contribute to the electrostatic interactions between water molecules and ions formed as a result of the dissociation of functional groups. In the case of each hydrogel composition, it is possible to observe that the sorption ability increases with time that is caused by the interactions between successive water molecules. In the case of swelling studies in liquids containing various ions, additional interactions take place. Therefore it was observed that slightly lower sorption ability was exhibited by sample 1. in SBF, Ringer’s liquid and artificial saliva compared to its swelling in distilled water. This may be an exact result of the presence of various ions in the mentioned swelling media. It was reported, e.g., by Feng et al. that the water sorption is lower when the concentration of ions in the swelling medium is higher [53]. As a result, the equilibrium swelling ratio in the simulated physiological fluids is lower than in distilled water. Ion presence in these solutions attributes to the relatively high osmotic pressure which limits to a certain extent the sorption properties of a sample immersed in liquids with the mentioned ions. Finally, penetration of a solution into the hydrogel material as well as the hydration of hydrophilic functional groups is limited in simulated physiological liquids and clearly lower than in the case when the swelling medium is distilled water [54]. Additionally, lower water sorption may be also a result of the fact that ions present in the swelling media affect the crosslinking density of the immersed hydrogel. For example, divalent cations (Ca^2+^) occurring, e.g., in Ringer liquid or SBF solution may crosslink the hydrogel structure through attaching to anions such as COO^−^ which presence is caused by the dissociation of COOH group in an aqueous environment. When a Ca^2+^ ion attaches to two neighboring COO^−^ ions, an additional crosslink is formed and hence an increase in the crosslink density of such hydrogel structure is observed. On the other hand, monovalent Na^+^ ions also negatively influence the sorption properties of the tested hydrogels. This is caused by the ion exchange between hydrogen ions and sodium ions. As a result of the neutralization reaction, the weakening of the hydrophilic nature of the -COOH group is observed.

Next, it may be observed that sample 0/3.2 exhibits lower sorption capacity than sample 0/1.6 in all tested liquids. Sample 0/3.2 was obtained using a higher amount of the crosslinker. This is a result of the fact that the crosslinking agent influences the crosslinking density of polymer chains that during the swelling process hydrate. Hence it may be concluded that the greater the crosslinking density of the hydrogel material, the lower its swelling ability. Similar conclusions were drawn by Zhang et al. They reported that different swelling abilities of hydrogels may be due to the differences in their microstructure. They conducted studies on the swelling capacity of hydrogels based on the methacrylated hyaluronic acid and containing additional co-monomer, i.e., acrylated bisphosphonate (Ac-BP). The addition of Ac-BP as a co-monomer resulted in the increase of the crosslinking density and therefore in the decrease of the swelling degree [55]. Such a conclusion is consistent with the results of the investigations presented in this paper. Next, the addition of the modifying agent, i.e., tea extract, causes a slight increase in the swelling ability. After the possible release of this additive from the tested material, the loosening followed by the separation of the polymer chains occurs and as a result, water and other physiological liquids have a larger possibility to penetrate the polymer network. This is a reason why such material is characterized by a slightly higher swelling ability. On the other hand, such a phenomenon may also be caused by the presence of numerous compounds in this additive which affect the osmotic pressure of the system and contribute to slightly more intense penetration of the swelling medium to the hydrogel material.

### 3.3. Results of Evaluation of the Mutual Interactions between Hydrogels and Simulated Physiological Liquids during Incubation Studies

**Figure 5:**
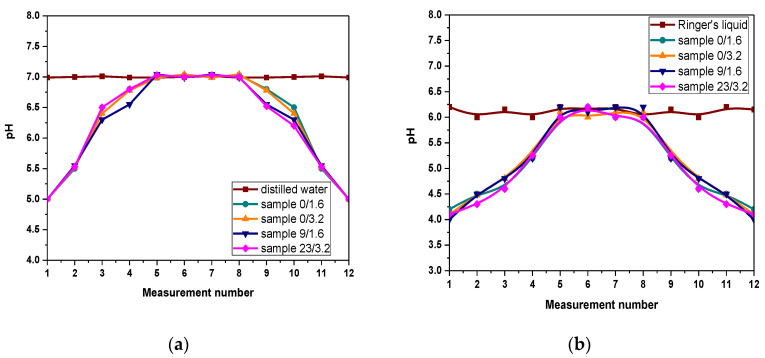

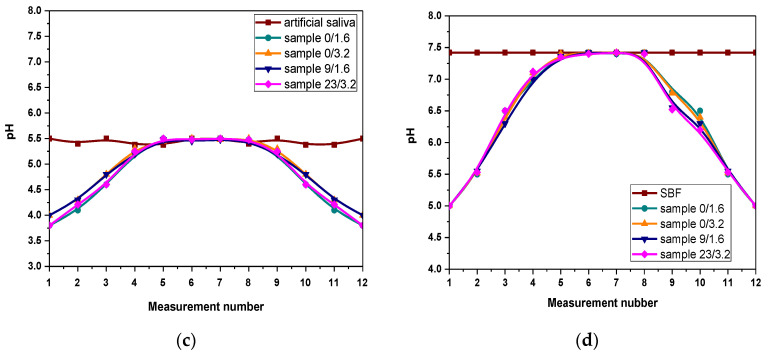
Measured pH values of distilled water (**a**); Ringer liquid (**b**); artificial saliva solution (**c**) and SBF (**d**) during incubation of hydrogel samples in these liquids.

The pH values of the tested liquids measured during the incubation studies performed are shown in Figure 5.

Based on the conducted investigations, a very similar course of curves illustrating the pH changes for all tested hydrogel samples subjected to the incubation tests may be observed. Tested materials were incubated in four different liquids, i.e., distilled water, SBF, Ringer’s liquid and artificial saliva. Furthermore, pH changes of these liquids without the sample immersed inside were also checked and treated as reference liquids. It may be observed that examined hydrogels have a significant impact on the pH of the tested liquids.

Firstly—i.e., for the first week of the research (first four measurements)—an increase in pH until its value will be equal to the value of these liquids without samples was observed. This is defined as the so-called buffering effect that takes place when the hydrogel begins to swell (absorb the incubation liquid). As a result, an increase in pH occurs. Due to the fact that these materials are characterized by buffering properties, the pH adjustment to the original value of the incubation liquid is observed. For the next week (next four measurements) pH maintains at the constant value. However, after a certain period (eighth pH measurement, i.e., after approx. two weeks of the immersion) a decrease in pH is observed. Such a sudden jump in the pH value (increase or decrease) after two weeks of incubation is usually related to the degradation of the tested material. Therefore it may be concluded that the degradation process of the immersed samples after two weeks of incubation takes place. It is also possible that the release of the modifying substance from the interior of the hydrogel matrix, i.e., tea extract, may cause the acidification of the incubation liquids and as a result, the pH decrease occurs. However, most probably is that such a process takes place when the material swells during the first week of the incubation and therefore the first few pH measurements indicate the relatively low pH of the incubation media. Additionally, chitosan was introduced into the reaction mixture in the form of its solution in an acetic acid so this environment may also affect the pH of the tested liquid in which chitosan-based hydrogels (both unmodified and modified ones) were incubated. On the other hand, the previously mentioned pH decrease observed after two weeks of immersion is caused probably by the degradation of the immersed sample. Thongchai et al. have also indicated the degradation process proceeding during the incubation studies. They conducted incubation studies of hydrogels based on chitosan and collagen in distilled water for 15 days. After only 3 days of the study, it was reported that the significant degradation of the tested material has occurred. Such results were confirmed by the hydrogel mass loss [56]. Additionally, such a conclusion is supported by the fact that analyzed hydrogels were synthesized using also gelatin and acetic acid which are characterized by pH < 7. Therefore, their release from the hydrogel as a result of its degradation may cause previously mentioned acidification. Such conclusions allow us to state that for the first week of incubation materials interact with the incubation media that leads finally to the pH stabilization for the next week of the immersion—hydrogels show their buffering properties and stability in tested environments. Nonetheless, after this time their probable degradation occurs. This, in turn, allows us to state that the developed materials may be effectively used in conditions simulating these occurring in the human body for two weeks.

### 3.4. Analysis of the Impact of Incubation in Simulated Body Liquids on the Chemical Structure of Hydrogels via FT-IR Technique

**Figure 6:**
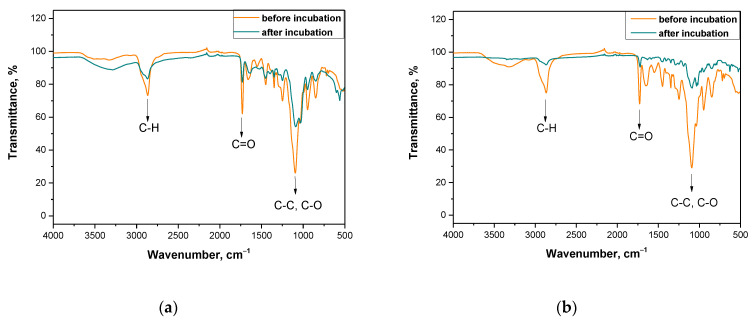
FT-IR spectra of (**a**) sample 0/1.6; (**b**) sample 0/3.2.

**Figure 7:**
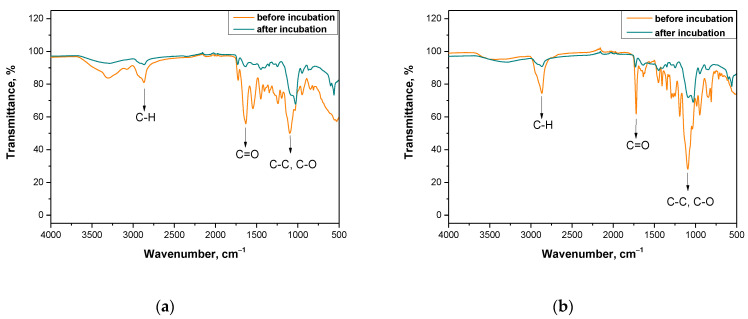
FT-IR spectra of (**a**) sample 9/1.6; (**b**) sample 23/3.2.

In order to evaluate the impact of the incubation on tested samples more carefully and to verify the conclusions drawn based on the pH changes, further research methodology included FT-IR spectroscopic analysis of hydrogel samples before and after the immersion in SBF solution. Obtained FT-IR spectra of the hydrogels are presented in Figure 6 and Figure 7.

The above-presented research was conducted for materials directly after the synthesis and for samples after the incubation studies (after three weeks of immersion in selected liquids). As it was mentioned previously—i.e., during the analysis of incubation investigations—it is supposed that after two weeks of the immersion of tested materials in physiological liquids their degradation occurs. As it may be seen on each spectrum of the sample after the incubation, the disappearance of some bands and the decrease of the band intensity of other peaks corresponding to the specific functional groups or bonds took place. The disappearance of some peaks or the decrease of the band intensity of the rest of the peaks corresponds to the degradation of the immersed sample that confirms previous conclusions reported based on the results of incubation studies. Functional groups or specific bonds that were marked in figures presenting the spectra are derived from chitosan, gelatin and the crosslinking agent, i.e., materials which are components of analyzed materials. However, any additional peak that could derive from the modifying substance—i.e., yellow tea extract—was not observed on the spectra. This is probably caused by too little amount of this additive in the hydrogel matrix or by the overlapping of peaks deriving from yellow tea extract and from other components of the tested polymer matrix. On the other hand, higher intensity of peaks deriving from samples with a larger amount of the crosslinker applied (i.e., Figure 6b and Figure 7b) is observed. The larger amount of crosslinker, the higher intensity of the peak on obtained spectra.

The methodology applied allowed us to verify the presence of characteristic functional groups in the structure of tested polymers before and after their incubation in SBF solution. This, in turn, allowed us to assess the impact of such an environment on the hydrogels and vice versa, that is the impact of hydrogels on such an incubation liquid. It was proved that after the incubation period a degradation of the tested materials occurs. However, any significant impact of the amount of the crosslinker or the yellow tea extract introduced into the polymer matrix on this process was not observed. Nonetheless, the results of this study are very satisfying and promising because on obtained spectra any additional peaks, that may indicate the formation of any unknown or undesirable substances as a result of the degradation process, were not observed. Moreover, the information concerning the degradability of the tested materials excludes the possible subsequent problem of their disposal that is a costly and problematic process.

### 3.5. Results of Studies on the Wetting Properties of Hydrogels

**Figure 8:**
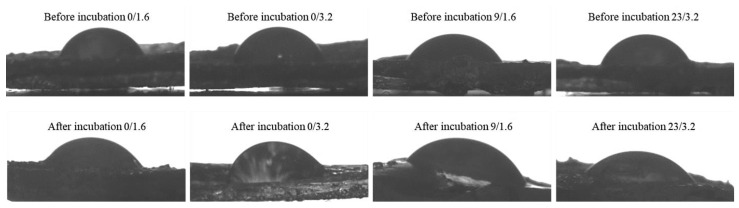
Results of the wettability analysis of samples before and after incubation.

The further step involved determining the wettability of hydrogel samples. The methodology applied allowed to provide information on the behavior of the drop of water dosed on the surface of the tested hydrogels as well as on the impact of the chemical compositions and pH of SBF solution on hydrogel wettability. Images of the drops of distilled water in contact with selected hydrogel samples with the wetting angles marked (one example image among three measurements performed) are presented in Figure 8. The study was performed for hydrogel materials differing in the content of crosslinker and yellow tea that were subjected to incubation in SBF. Measurement was repeated three times for each type of sample (*n* = 3).

In Table 2. average values of measured contact angles are given.

The methodology of the measurements allowed us to determine the wettability of hydrogel samples before and after incubation in SBF solution. Furthermore, the selected method enabled us to present the first contact of a drop of water with the analyzed sample’s surface both graphically and numerically (as a value of the contact angle). Firstly, it may be observed that incubation of hydrogels in simulated physiological liquid clearly affected the contact angles of tested polymers wherein a more significant impact was observed in the case of modified hydrogels, i.e., samples 9/1.6 and 23/3.2. Next, samples obtained using a higher amount of the crosslinker exhibited higher values of contact angles, i.e., lower wettability. It is a result of the fact that these samples have a very compact structure (with higher crosslinking degree) and a corrugated surface. Then, penetration of water into such a structure is limited because drops of water remain on such surface folds and as a result, water drops spill over the surface of such sample to a lesser extent. Next, modified samples are characterized by higher wettability, which means that water drops spill over their surface to a higher extent. The structure of modified samples is not as compact as these of unmodified ones and a surface is less corrugated therefore lower contact angles are observed.

**Figure 9:**
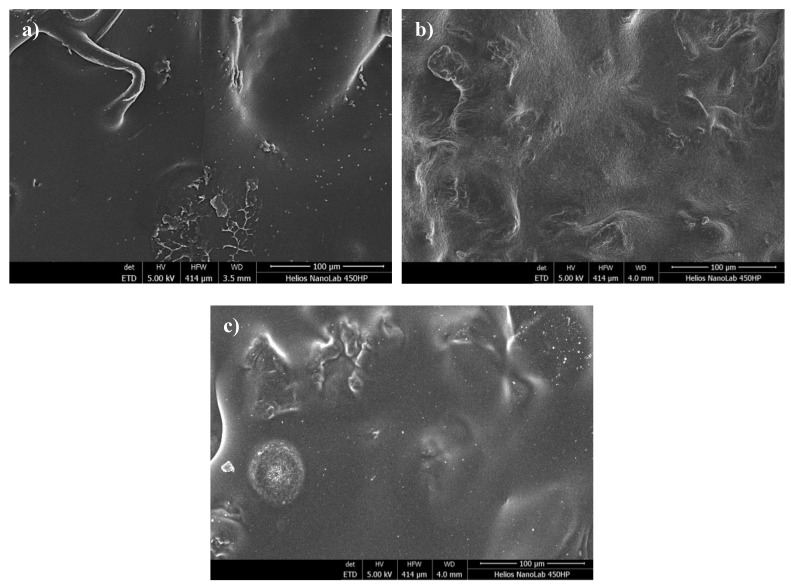
SEM images of unmodified hydrogel samples based on (**a**) Beetosan^®^ and (**b**) commercial chitosan and (**c**) sample modified with yellow tea extract (sample 23/3.2).

Next, it may be observed that the materials before the incubation period are characterized by a significantly larger contact angle, e.g., 89.2 ± 1.7° and 90.3 ± 0.9°, than samples after this study. The material after the incubation period is characterized by a definitely lower surface wetting angle, i.e., in the case of the sample prepared using a lower amount of the crosslinker and the yellow tea extract this angle is 70.2 ± 1.4° and in the case of the sample with higher amount of the crosslinker and the yellow tea extract—5.0 ± 2.1°. The decrease of the surface wetting angle after the incubation period is caused probably by the degradation process occurring as a result of which the external structure of the analyzed materials becomes less homogeneous and compact. (Figure 9a). Therefore, the drop spills over the tested material and thus the contact angle is significantly lower. Considering the difference between the surface wetting angles of sample 9/1.6 and sample 23/3.2 after the incubation period, this is probably caused by the different compositions of these hydrogel matrices. The larger amount of yellow tea extract in the composition of sample 4. affects that its wetting angle is lower than in the case of sample 9/1.6. This is probably caused by the elution of the tea extract from the interior of the tested material and thus a greater loosening (“opening”) of the polymer network. Then, free spaces between polymer chains are larger and the applied drop of water spills over the material to a greater extent and the wetting angle is lower.

The application of various modifying agents brings different effects. For comparison, Khorasani et al. performed investigations on hydrogels based on chitosan and poly(vinyl alcohol) PVA and modified with zinc oxide nanoparticles. They proved that the addition of ZnO nanoparticles caused the increase in the hydrophobicity of such modified hydrogels and thus resulted in the reduced ability of the surface of such material to absorb water and finally in the increased contact angle [57].

Another explanation of the lower contact angle of sample 23/3.2 compared to sample 9/1.6 is a greater amount of the modifier used for the synthesis of sample 23/3.2. In the composition of the yellow tea, there are many compounds with hydrophilic functional groups which in contact with water may form hydrogen bonds. This, in turn, results in more intense attraction and spilling the drop over the surface of the sample causing the decrease of the wetting angle.

### 3.6. SEM Analysis of Hydrogel Surfaces

Images of analyzed hydrogels obtained via scanning electron microscopy are shown in Figure 9.

The application of the SEM technique allowed us to note the differences between a sample of chitosan-based hydrogel obtained from naturally deceased bees and a sample obtained using commercial chitosan. Both analyzed samples were not modified with yellow tea. The structure of both materials is very compact, which indicates high cross-linking of samples. This is due to the use of a large amount of crosslinking agent during the synthesis. The Beetosan^®^-based sample did not show any significant differences in its structure compared to the hydrogel based on commercial chitosan. This proves that the material obtained from honeybees, used for the preparation of hydrogels, was previously properly deprived of other substances such as proteins or waxes. Microphotographs of the tested materials are presented in different magnifications, however, it can be concluded that hydrogel matrices based on Beetosan^®^ are almost identical to those ones obtained using commercial chitosan. Therefore, the preparation of chitosan from a new source, such as honeybees, is becoming more and more widespread. Additionally, it may also be concluded that the modification of hydrogel with yellow tea extract did not affect the surface morphology of obtained samples so that such an additive may provide the material with new, therapeutic features without affecting its properties.

### 3.7. Results of the Investigations on the Tensile Strength of Hydrogels

**Figure 10:**
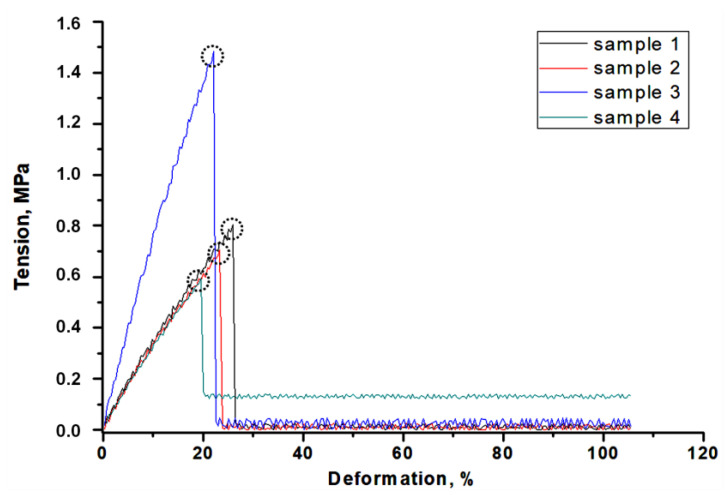
The scheme of the tensile strength analysis.

The measurement methodology based on determining the elongation ability of hydrogels under the tension applied using the texture analyzer provided information on the tensile strength of the hydrogels, their elasticity and the meaningless of such characteristics as the chemical composition or the crosslinking degree in the evaluation of the mechanical properties of hydrogels. In Figure 10. the scheme of the desired results of tensile strengths showing the dependence between the deformation of the tested sample under the tension applied (wherein the circles show the tension under which the sample was broken) is shown.

**Figure 11:**
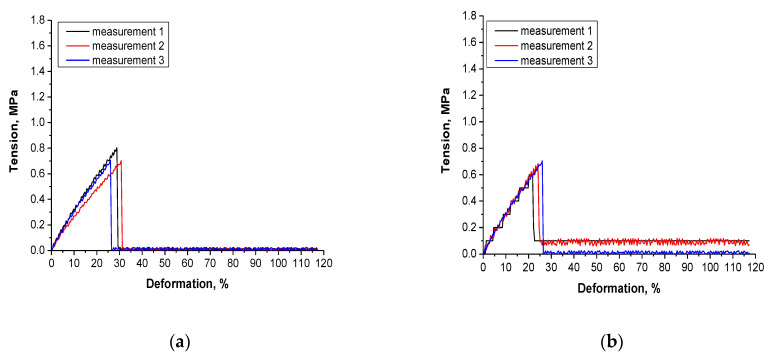

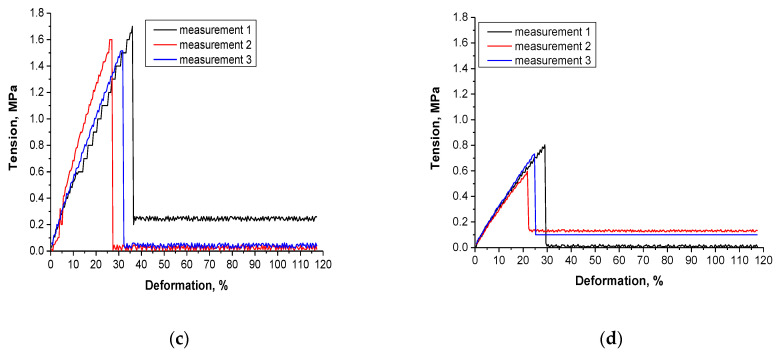
Results of tensile strength analysis of sample 0/1.6 (**a**), 0/3.2 (**b**), 9/1.6 (**c**) and 23/3.2 (**d**).

The results of the mechanical measurements are presented in Figure 11.

Deformation of hydrogel samples under selected tension as well as maximum deformation under which sample has broken are indicated in Table 3.

The performed investigation and the methodology applied allow us to determine the tensile strength of the materials and at the same time characterize the impact of the addition of the tea extract and the amount of the crosslinker on this property. Based on the conducted analysis it may be observed that sample 9/1.6 exhibited the most favorable strength properties. This is a sample that contains lower amounts of crosslinker and tea extract than sample 23/3.2. Other materials are characterized by similar strength properties but exactly sample 23/3.2—i.e., this one prepared using the greater amounts of the crosslinker and the tea extract—exhibits the least favorable strength properties. Therefore it may be concluded that the addition of the tea extract depending on its amount may significantly improve or worsen the strength properties of prepared hydrogel materials. Despite the fact that the amount of the crosslinker also constitutes a variable, the difference between the strength properties of sample 0/3.2 and sample 9/1.6 is similar. Therefore it may be stated that exactly the addition of the tea extract caused the improvement or the deterioration of the mechanical properties. Too large an amount of the yellow tea extract may cause the loosening of the polymer network or insufficient crosslinking of the material that results in the poor tensile strength of the analyzed material. Such conclusions are analogous to these ones drawn by Chen et al. They presented the differences in the mechanical strength of hydrogels obtained using different concentrations of gelatin and polyacrylamide. Based on the conducted studies they conclude that the type of the crosslinker but also, importantly, mass fractions of reagents applied have an impact on the mechanical properties of the hydrogels [58].

## 4. Conclusions

The proper course of the crosslinking of hydrogels requires an adequate selection of all applied reagents used for the synthesis of such materials. In the case of an inadequate proportion of the reagents applied, the crosslinking may not occur at all or may be incomplete.

Beetosan^®^-based hydrogels degraded after two weeks of immersion in physiological liquids as evidenced by the sudden pH decrease noted on the eighth day of the incubation period (e.g., from 6.00 to 5.25). Their degradation was proved via a comparison of samples’ structure before and after incubation using FT-IR analysis. Yellow tea extract present in hydrogel matrices did not affect this process. 

Incubation of hydrogels in simulated body fluid affected their wettability. Samples after this study exhibited lower surface wetting angles, a finding which was likely related to the degradation process.

Hydrogels exhibited a high swelling capacity, i.e., even 3.0–3.5 g/g. This property depended on some factors, i.e., the higher amount of the crosslinker applied during the synthesis, the lower the swelling ability and the higher amount of yellow tea extract, the higher the sorption capacity of such modified hydrogel.

Yellow tea extract introduced into the hydrogel matrix affected the tensile strength of the hydrogels. A too-large amount of this additive, i.e., 23% v/v, adversely influenced the mechanical strength of such modified hydrogels via too intense dilution of the reaction mixture and probably inadequately crosslinked hydrogel obtained. On the other hand, 9% *v/v* of this extract resulted in an increase in the elasticity of the hydrogels.

Physicochemical properties of prepared hydrogels define them as promising materials with application potential for use for biomedical purposes, e.g., as innovative dressing materials which absorb the wound exudate, exhibit elasticity and provide additional therapeutic properties due to the presence of yellow tea extract in its structure. The next planned investigations involve the analysis of cytotoxicity of the materials using selected cell lines and the study of their impact on the wound healing process.

## Figures and Tables

**Table 1:** Compositions of synthesized hydrogels.

No.	Base Solution, mL	Yellow Tea Extract, % (*v*/*v*)	Crosslinking Agent, mL	Photoinitiator, mL	Sample Notation
**1.**	10	0	1.6	0.1	0/1.6
**2.**		0	3.2		0/3.2
**3.**		9	1.6		9/1.6
**4.**		23	3.2		23/3.2
**5.**		33	3.2		33/3.2

**Table 2:** Surface wetting angles of hydrogels.

Sample	Surface Wetting Angle, °	
	Before Incubation	After Incubation
0/1.6	91.7 ± 2.8	85.5 ± 1.2
0/3.2	93.5 ± 1.3	87.7 ± 2.3
9/1.6	89.2 ± 1.7	70.2 ± 1.4
23/3.2	90.3 ± 0.9	55.0 ± 2.1

**Table 3:** Deformation of samples under selected tension and the maximum deformations.

Sample	Deformation under 0.6 MPa Tension, %	Maximum Deformation, %
0/1.6	19	27
0/3.2	21	24
9/1.6	8	23
23/3.2	20	20

## Data Availability

The data presented in this study are available on request from the corresponding authors.
